# Dataset of real time multi-parameter monitoring for loess slopes in Gaolan mountain, Lanzhou, China: Multi-sensor network for hydrology and geophysics

**DOI:** 10.1016/j.dib.2025.111943

**Published:** 2025-08-05

**Authors:** Shuangshuang Li, Peng Han, Yihua Zhang, Shuangling Mo, Jianwei Sun, Yuyan Li, Kaiyan Hu, Gexue Bai, Ruidong Li, Baofeng Wan, Guoxuan Ding, Bingbing Han, Fangjun Li, Fanyu Zhang

**Affiliations:** a Department of Earth and Space Sciences, Southern University of Science and Technology, Shenzhen 518055, China; b Changchun Institute of Optics, Fine Mechanics and Physics, Chinese Academy of Sciences, Changchun 130033, China; c School of Geophysics and Geomatics, China University of Geosciences (Wuhan), Wuhan 430074, China; d Gansu Institute of Engineering Geology, Lanzhou 730000, China; e College of Civil Engineering and Mechanics, Lanzhou University, Lanzhou 730000, China

**Keywords:** Landslide, Field experimental site, In-situ geophysical data, Loess plateau, China

## Abstract

Landslides pose significant threats to human life and infrastructure globally. In China, the intensification of urbanization and human activities has exacerbated loess landslide risks, making monitoring and mitigation efforts increasingly critical. Rainfall, surface displacement, pore pressure, and seismic waves as key parameters for landslide monitoring. Therefore, a real time multi-sensor observation system was deployed in July 2019 at the Gaolan Mountain (36°01′10.4″N, 103°50′52.4″E) experimental site in Lanzhou, China, a region prone to frequent landslides. This dataset provides ​high-frequency measurements of hydrological and geophysical parameters, offering a comprehensive view of landslide dynamics. Preliminary results demonstrate the ​accuracy and reliability of the system, highlighting its potential to enhance landslide early warning capabilities. The dataset is publicly available under DOI: [10.5281/zenodo.15180387], supporting further research on slope water infiltration and hazard risk management in loess regions.

Specifications Table

**Table:** 

Subject	Earth & Environmental Sciences
Specific subject area	Monitoring and early warning of Loess disasters, hydrological and geophysical responses during the rainfall process.
Type of data	Raw data: Monitoring data (.dat), LiDAR point cloud (.las) Processed data: Digital Elevation Model (.tif)
Data collection	Field monitoring site for loess disaster was established in the Gaolan mountain are of Lanzhou, China, recording from August 2019 to March 2020. A total of 3 uniaxial accelerometers, 1 triaxial accelerometer; 16 self-potential electrodes along the sliding direction (profile length: 16 m); 5m-deep pit with temperature-moisture content sensors at 1-m intervals, 1 pore pressure sensor at 5 m depth of pit; 1 wire extensometer at the upper slope; Rainfall, light, and wind sensors at the mid-slope were deployed (see Fig. 2). The terrain data of this station was obtained in July 2024 using a DJI M300 RTK equipped with L1 laser radar scanning. Through applying 4G internet of things technology, meteorological data that trigger disasters and hydrological and geophysical response data during the disaster process can be obtained in real time and stored.
Data source location	Gaolan mountain, Lanzhou, Gansu province, China 36°01′10.4″N, 103°50′52.4″E (WGS84, EPSG:4326)
Data accessibility	Repository name: Monitoring Dataset of Loess Slopes in Gaolan Mountain(MDLS-GLM) Data identification number: 10.5281/zenodo.15180387 Direct URL to data: https://zenodo.org/records/15,180,387
Related research article	None

## 1 Value of the Data

• The dataset integrates ​geophysical, ​meteorological, and hydrological data, providing a holistic view of landslide dynamics in loess slopes. This multi-source approach enhances the understanding of landslide triggers and mechanisms.
• This dataset recorded self-potential and accelerometer data during landslide processes, providing a valuable reference for the practical application of geophysical methods in landslide monitoring and early warning.
• As a product of field experiments, this dataset provides long-term monitoring data from natural loess slopes, offering valuable empirical support for laboratory studies and theoretical models. Such field-based observations enhance the reliability of landslide research by bridging the gap between experimental simulations and real-world slope behavior.
• The dataset was collected from the loess region of Gola Mountain in Lanzhou, China, a region characterized by distinctive geological and climatic conditions. Researchers in climatology, environmental science, and geohazard studies can leverage this dataset to advance disaster risk assessment and mitigation strategies.

## 2 Background

China is among the countries most severely affected by landslides globally, with loess-covered regions experiencing particularly high susceptibility [1]. Consequently, the development of effective landslide monitoring and early warning systems has become a critical component in disaster mitigation and risk management strategies. Hydrological processes triggered by rainwater infiltration have been widely recognized as a predominant mechanism for landslide initiation, with critical parameters including volumetric water content, pore water pressure, and slope displacement serving as essential indicators for landslide early warning systems [[2], [3], [4], [5]]. Geophysical methods have demonstrated significant efficacy in capturing the dynamics of rainwater infiltration within slopes and the progressive rupture processes in sliding zones [[6], [7], [8], [9], [10]], thereby substantially enhancing the capabilities of landslide monitoring and early warning systems.

To investigate the interaction mechanisms among multiple parameters during the evolution of loess landslides and to identify practical criteria for early warning. A real-time monitoring system based on multiple sensors was deployed in July 2019 at the experimental site in Gaolan Mountain, Lanzhou, China. This system is designed to collect meteorological data (e.g., rainfall, light intensity, wind speed), hydrological data (e.g., soil temperature, moisture, pore pressure), and geophysical observation indicators (e.g., accelerometer, self-potential, slope displacement) during the loess landslide evolution process.

## 3 Data Description

The dataset consists of three main subsets: meteorological, hydrological, and geophysical data, each capturing different aspects of loess slope stability at the Gaolan Mountain experimental site in Lanzhou, China. Time-series monitoring data files are named according to the format YYYY_MM_DD_HH_MM_SS​​ to indicate the year, month, day, hour, minute, and second of recording. Each hourly record is stored as an individual ​​ YYYYMMDDHHMMSS.dat​​ file (approximately 49.2 M), while monthly data are organized into separate folders and compressed (YYMM.7z). The measured physical quantities associated with each data column are specified in the file (Read_me.jpg).

Meteorological data: comprises time-series records of rainfall intensity (mm/h), wind speed (m/s), and solar radiation (W/m²), which facilitate the examination of external environmental factors influencing slope instability.

 Hydrological data: hydrological measurements were obtained from five depth layers (0.5–5 m), recording soil temperature (°C), volumetric water content (%), and pore water pressure (kPa). These data enable comprehensive analysis of subsurface hydrological processes.

Geophysical data: synchronized monitoring records include three-dimensional acceleration (g), self-potential (mV), and displacement (mm), providing essential parameters for assessing slope deformation characteristics and internal mechanical responses.

In addition to the monitoring data, topographic data were acquired, consisting of UAV-derived point cloud data (.las format) and processed digital elevation models (.tif format). These data provide detailed spatial characterization of the monitoring site. The dataset also contains annotations marking abnormal data periods, particularly sensor malfunctions occurring during heavy rainfall events in August 2021, thereby ensuring data transparency and reliability for subsequent analyses.

## 4 Experimental Design, Materials and Methods

### 4.1 Study site

**Fig. 1:**
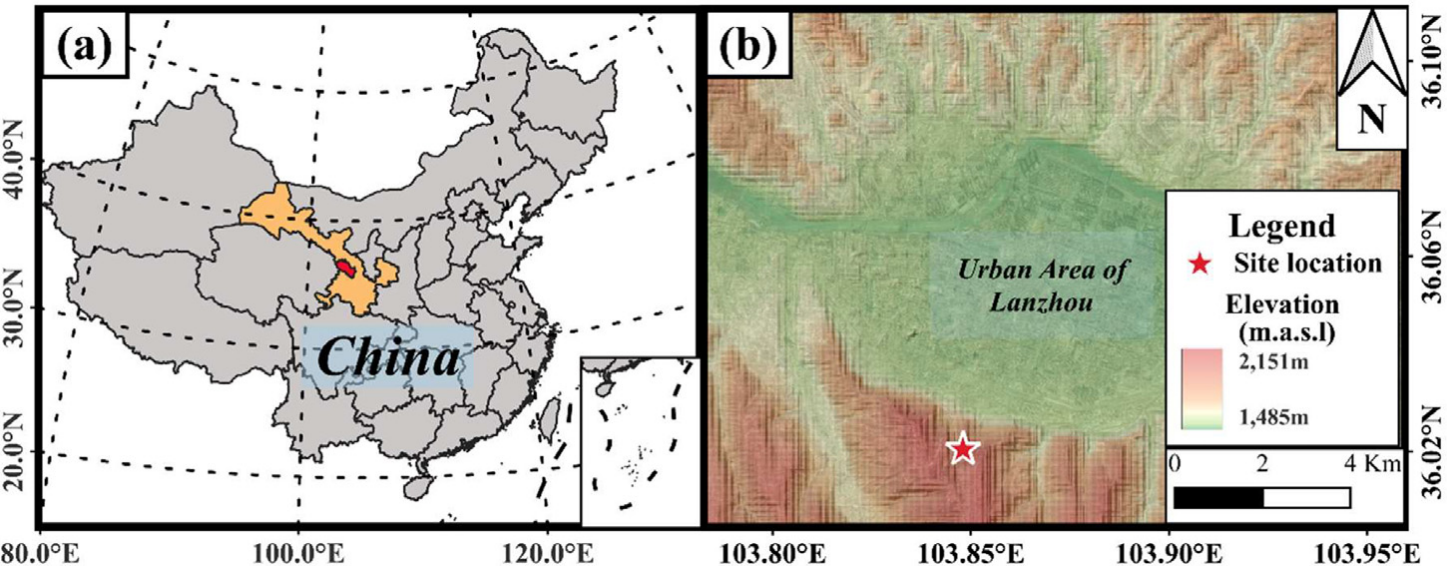
Map of the study area: (a) Location of Lanzhou (highlighted in red); (b) Location of field monitoring sites on a hillshaded digital elevation model (marked with red stars).

The Gaolan Mountain is located south of Lanzhou's urban area and north of Chengguan District's Ethnic Village (Fig. 1), with geographical coordinates of 103.84°E, 36.03°N (WGS84). The largest gully in this area is Laolang Gully, whose mouth connects to Lanzhou Railway Station and the downtown area. As a typical loess gully, Laolang Gully has significant topographic undulations, steep slopes, low vegetation coverage and loose debris in the gully, which create favorable conditions for the occurrence of landslides and collapses [11]. Preliminary field investigations identified 91 landslides within the gully, comprising 49 rotational slides, 37 translational slides, and 5 collapses [[12], [13]]. The experimental site represents a translational landslide located on the eastern flank of the gully, characterized by an average slope angle of 35°. Field observations confirm active slope movement, which establishes this location as an ideal natural laboratory for landslide mechanism studies.

### 4.2 Sensor network deployment

**Fig. 2:**
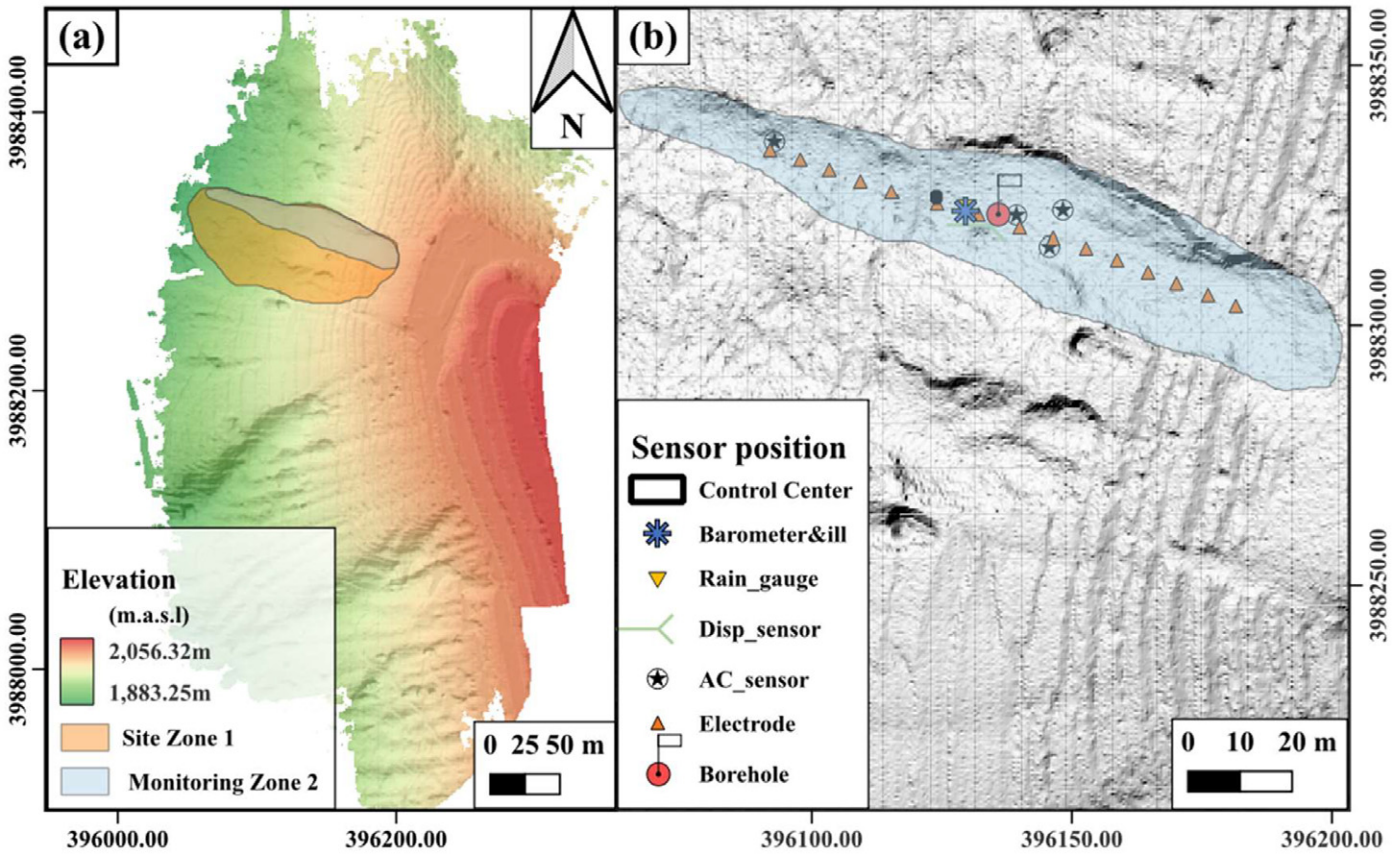
UAV radar topographic map of the experimental site: (a) Digital elevation terrain and slope sliding range and (b) Main monitoring area and sensor deployment locations.

In July 2024, unmanned aerial vehicle (UAV) LiDAR surveys were performed in the study area using a DJI M300 RTK drone equipped with a Zenmuse L1 LiDAR sensor to acquire high-resolution topographic data. The resulting point cloud dataset achieved approximately 5 cm accuracy, referenced to the CGCS2000 coordinate system (3-degree zone 105E). Following point cloud classification and vegetation removal, the processed digital elevation model (DEM) is shown in Fig. 2a, revealing an 80 m elevation difference between the front and rear of the experimental site. Monitoring sensors were installed along the slope's left flank, with their precise coordinates displayed in Fig. 2b.

**Fig. 3:**
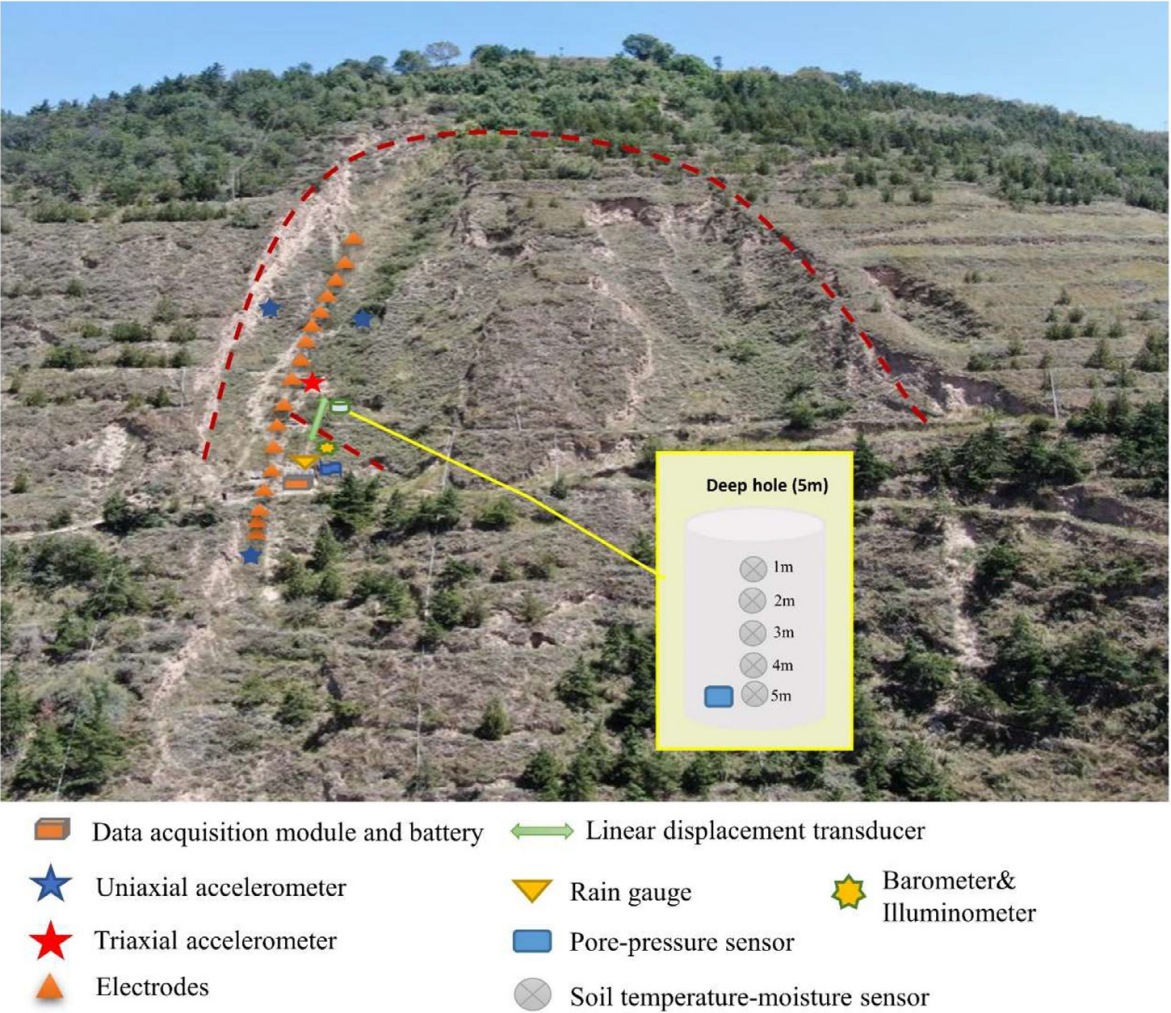
Sensor distribution at the experimental site (red dashed line indicates the sliding area).

An aerial view of the deployment of the sensor network and the data acquisition/transmission system is illustrated in Fig. 3. The system is powered by three parallel lithium-ion battery packs (total capacity: 360 Ah), which are recharged by solar panels via a power controller. The sensor array consists of three uniaxial accelerometers, one triaxial accelerometer, one displacement transducer, one rain gauge, one illuminometer, one barometer, multiple electrodes, pore-pressure gauges, and soil temperature/moisture sensors. Complete sensor specifications are listed in Table 1.

**Table 1:** Detailed information of each sensor.

Sensor category	Monitoring physical quantity	Main properties	Sampling frequency
Pore pressure sensor	Pore pressure	Type: KPA-200KPA Capacity:200Kpa Rated output:754uv/V Non-linearity:0.3 %RO	10HZ
Uniaxial accelerometers	Acceleration	Type: CA-YD-189 Sensitivity: 98.8mv/(m*s^2^) Frequency range: 0.2–1000Hz Maximum allowable acceleration: 5g	200HZ
Tri-axial acceleration	Acceleration	Type: CA-YD-3100 Sensitivity: 50mv/(m*s^2^) Frequency range: 0.2–500Hz Maximum allowable acceleration: 1000g	200HZ
Barometer& Illuminometer	Air pressure& Illumination intensity	Type: RS-QY-*–2–4 & RS-GZ-*-2 Range: 0∼120Kpa & 0–65535Lux Voltage output: 0–5V	10HZ
Displacement transducer	Displacement	Type: MPS-M-3000mm Range: 0–3000mm Voltage output: 0–5V Accuracy: ±0.03 %FS	10HZ
Soil temperature and moisture	Temperature, volumetric water content	Type: NHSF48BU Principle: Frequency-Domain Reflectometry (FDR) Voltage output: 0–2V Accuracy: ±3 %	10HZ
Rain gauge	Precipitation	Type: HC-YL9072 Range: 0.01mm-4mm/min Voltage output: 0–5V Accuracy: ±0.1mm	10HZ
Electrode	Electric potential	Type: PMS9000 Polarization potential difference: <0.1mV Temperature response: 100µV/ °C Internal resistance: <500Ω	10HZ

Considering the maximum frozen soil depth of approximately 1.2 m in this region [14], all electrodes were installed at 1.5 m depth. Seventeen electrodes were deployed, with the uppermost electrode designated as the reference. Potential differences between each electrode and the reference electrode were recorded simultaneously. Soil temperature moisture sensors are installed at intervals of 1 m in 5-meter-deep boreholes, and there is also a pore pressure sensor at the bottom (see the inset in Fig. 3). The triaxial accelerometer was positioned at the slope's midpoint, while two uniaxial accelerometers were placed at the top and one at the base. The displacement transducer was installed within the stepped crack zone to monitor either relative displacement between two points or landslide settlement. The rain gauge, barometer, and illuminometer were co-located near the solar panel array.

### 4.3 Data acquisition protocol

The monitoring system consists of three core components: a signal acquisition module; a data visualization module, and a data storage and transmission module. The system employs GPS time synchronization technology with sub-millisecond precision (<1 ms) to ensure temporal alignment across all sensors. A LabVIEW-based acquisition program, integrated with National Instruments (NI) cDAQ devices, facilitates real-time multi-channel data acquisition with adaptive sampling rates optimized for each sensor type.

Raw data processing follows two parallel pathways:

1. Unprocessed datasets are stored locally on hard drives at full resolution
2. Raw data undergo median filtering and downsampling to 1/60 Hz before encryption for secure transmission via 4G/5G networks

**Fig. 4:**
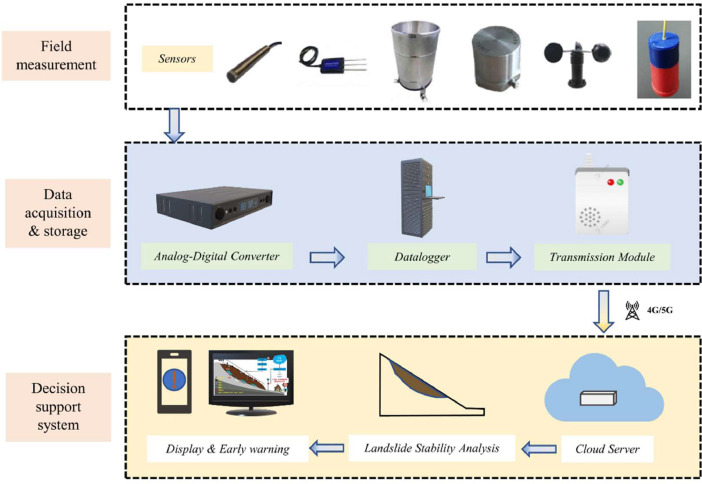
Schematic diagram of the landslide data acquisition, monitoring, and early warning system.

The processed data are visualized through both an interactive web dashboard and mobile application. This dual-architecture approach (Fig. 4) combines local storage with cloud analytics to ensure both data security and accessibility. The MySQL database implements version-controlled backups through daily snapshots and binary logging, maintaining data integrity throughout the 30-day retention period.

### 4.4 Sample of data

**Fig. 5:**
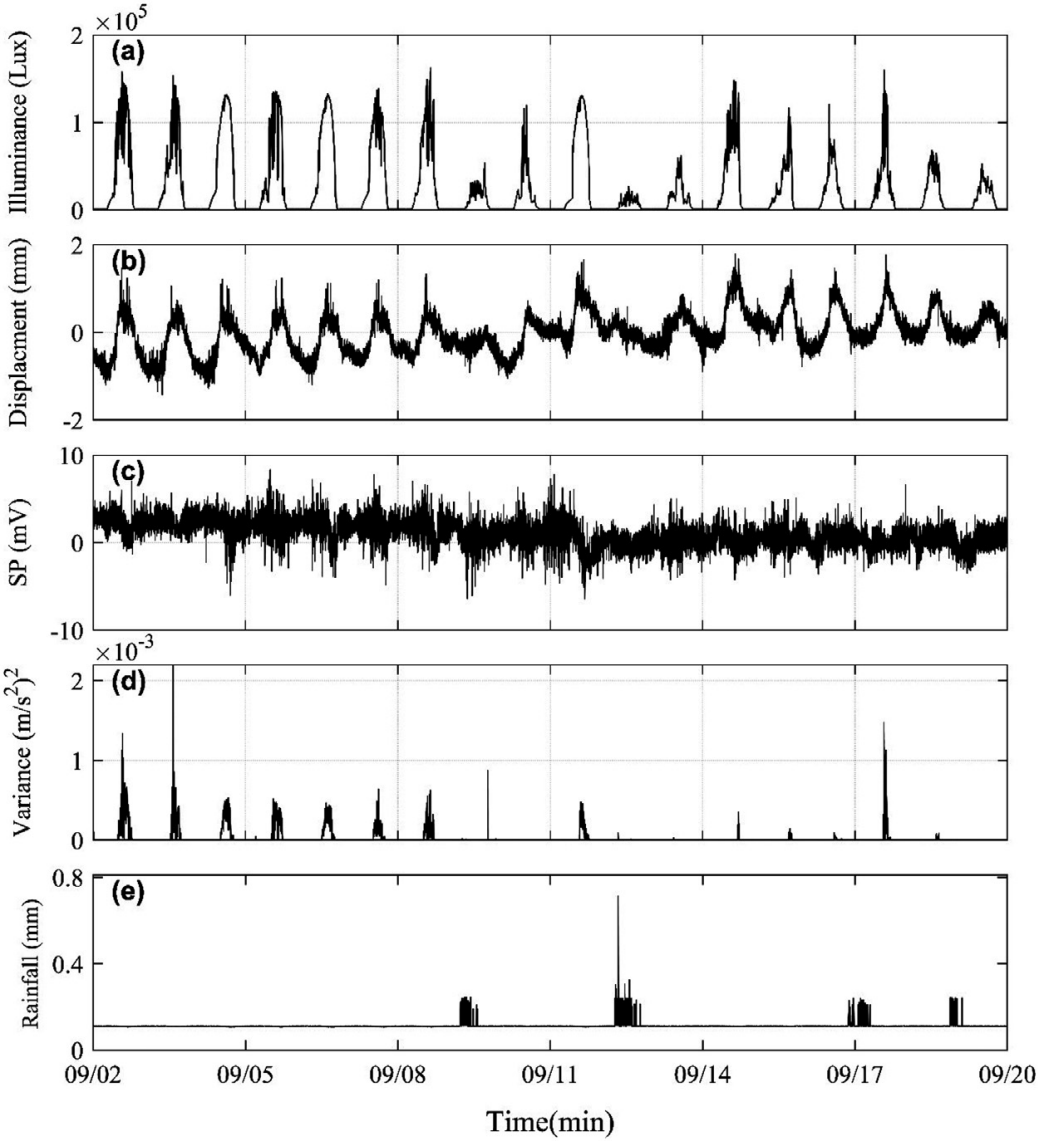
Monitoring results of illuminance, surface displacement, self-potential, acceleration, and rainfall from September 2 to 20, 2019 (sampling interval: 1 min).

To present the data more clearly, a portion of the data from September 2 to 20, 2019 was extracted as an example. Figs. 5 presents the integrated monitoring dataset, comprising illuminance, displacement, self-potential, acceleration, and rainfall measurements. Fig 5a shows that illuminance exhibits distinct diurnal variations, with solar radiation peaking at approximately 15:00 local time and reaching its minimum around 03:00. The displacement measurements, which record internal cable length changes. As indicated in Fig 5b, the displacement data represent deviations from the initial reference length (2700 mm).

Comparative analysis with rainfall data (Fig. 5e) demonstrates that both displacement and illuminance show attenuated diurnal variation amplitudes during precipitation events. The self-potential measurements acquired from the summit electrode array (Fig. 5c) reveal stable electromagnetic background noise throughout the observation period, with potential variations limited to several millivolts, demonstrating electrode stability.

Fig. 5d shows the variance of acceleration data recorded by a uniaxial accelerometer installed at the slope toe (original sampling rate: 200 Hz). During transmission, the data were downsampled to one averaged value per minute. During dry periods, the variance peaks consistently at noon. Field investigations identified these anomalies as being caused by pumping activities near the sensor location during midday hours. Following rainfall events, reduced or absent pumping activity corresponds to significantly diminished sensor response.

**Fig. 6:**
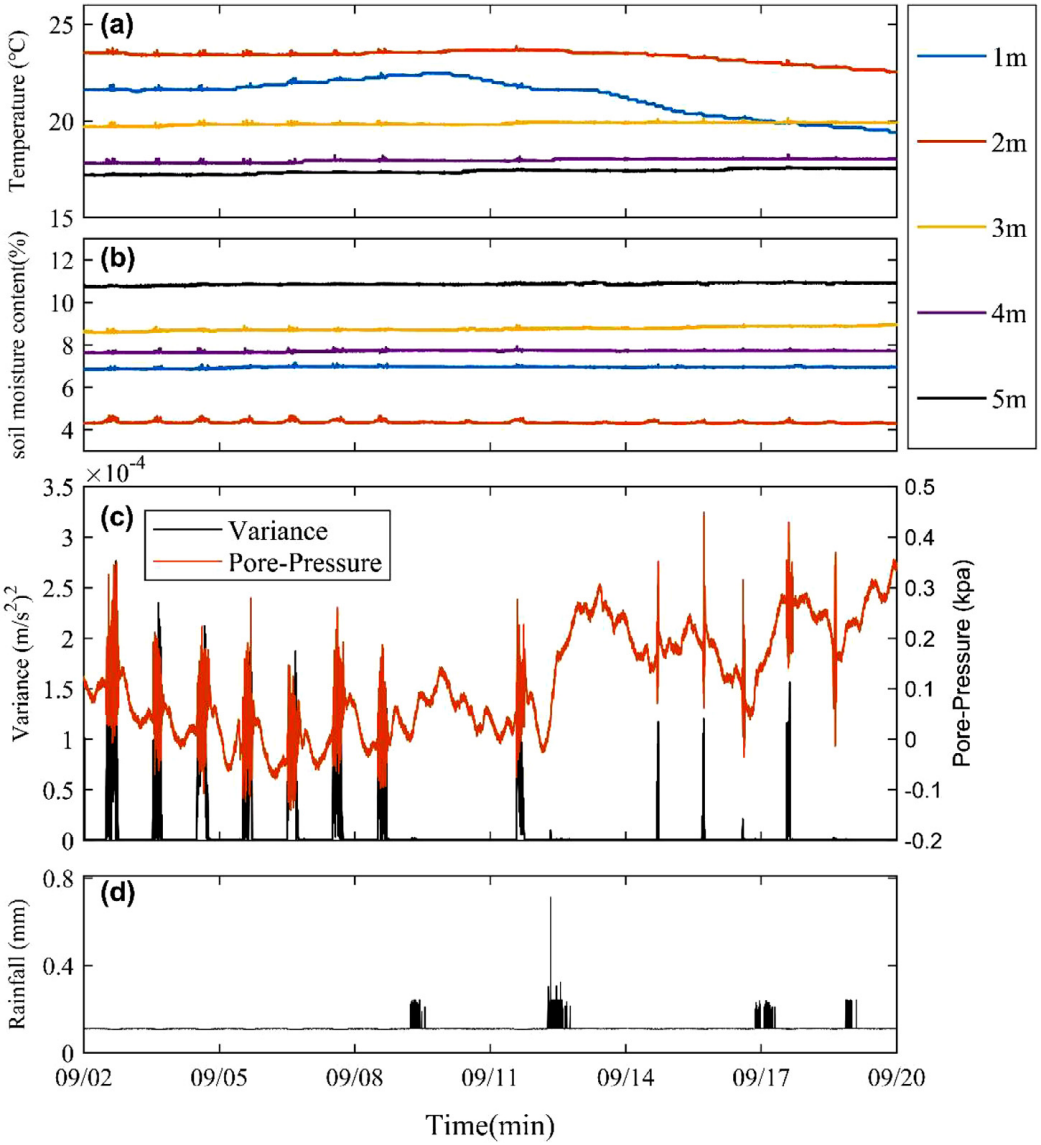
Time-series monitoring of in-situ borehole parameters from September 2 to 20, 2019 (1-minute sampling interval): (a) Depth-dependent temperature variations; (b) Depth-specific soil moisture content profiles; (c) Co-variation of acceleration variance (left axis) and pore-water pressure (right axis); (d) Rainfall intensity.

The measurements in the borehole are shown in Fig. 6. including temperature profiles, soil moisture content, pore-water pressure distribution, along with acceleration and rainfall. As shown in Fig. 6a, the rainfall event affected the shallow temperatures (at 1 and 2 m), while the temperatures at greater depths (3, 4, and 5 m) remained basically stable. The soil moisture content maintained remarkable stability across all monitored depths throughout the observation period. Fig. 6c displays the pore pressure and acceleration variance data at 5 m depth. On-site observations indicated that there were pumping irrigation activities above the slope top during the period of water shortage.

## Limitations

The acquisition of high-frequency geophysical data (e.g., acceleration measurements) generates large file sizes, which consequently require extended processing time for decompression and file access.

## Ethics Statement

The authors have read and follow the ethical requirements for publication in Data in Brief and confirm that the current work does not involve human subjects, animal experiments, or any data collected from social media platforms.

## CRediT authorship contribution statement

**Shuangshuang Li:** Investigation, Data curation, Writing – original draft, Writing – review & editing, Visualization. **Peng Han:** Conceptualization, Methodology, Investigation, Supervision, Project administration, Funding acquisition. **Yihua Zhang:** Investigation, Data curation, Software, Visualization, Writing – original draft. **Shuangling Mo:** Data curation. **Jianwei Sun:** Methodology, Data curation, Software. **Yuyan Li:** Software, Visualization. **Kaiyan Hu:** Methodology, Investigation. **Gexue Bai:** Supervision, Project administration. **Ruidong Li:** Supervision, Project administration. **Baofeng Wan:** Supervision, Project administration. **Guoxuan Ding:** Supervision, Project administration. **Bingbing Han:** Supervision, Project administration. **Fangjun Li:** Supervision, Project administration. **Fanyu Zhang:** Methodology, Investigation.

## Data Availability

Earth/ChemMonitoring Dataset of Loess Slopes in Gaolan Mountain(MDLS-GLM) (Original data).

## Appendix Supplementary materials

Image, application 1
